# Pterostilbene administration improves the recovery potential of extremely low-frequency magnetic field in acute renal ischemia-reperfusion injury: an FTIR spectroscopic study

**DOI:** 10.3906/biy-1907-18

**Published:** 2020-02-17

**Authors:** Hatice KESER, Özlem BOZKURT GİRİT*, Muhammed MAJEED, Mahadeva NAYAK, Mehmet Dinçer BİLGİN

**Affiliations:** 1 Department of Biophysics, Institute of Health Sciences, Aydın Adnan Menderes University, Aydın Turkey; 2 Department of Biophysics, School of Medicine, Karadeniz Technical University, Trabzon Turkey; 3 Department of Biophysics, School of Medicine, Aydın Adnan Menderes University, Aydın Turkey; 4 Sabinsa Corporation, East Windsor, NJ USA; 5 Technical Marketing, Sami Labs Limited, Bangalore, Karnataka India

**Keywords:** Renal ischemia-reperfusion injury, extremely low-frequency magnetic field, pterostilbene, Fourier transform infrared spectroscopy

## Abstract

Renal ischemia-reperfusion (I/R) injury, one of the drastic outcomes of renal failure and organ transplantation, tends to deteriorate over time; therefore, noninvasive therapeutic strategies will avail the progression-free survival of the patients. Magnetic field has been proposed as a noninvasive treatment strategy; however, with recent scientific advances, many controversies have arisen regarding its efficacy. Pterostilbene, a natural analog of resveratrol, was documented to be effective in treatment of I/R injuries. This study aims to assess the acute therapeutic effects of combined extremely low-frequency magnetic field (ELF-MF) and pterostilbene treatment on renal I/R injury. After induction of renal I/R in Wistar rats, treatments of 50 Hz, 1 mT ELF-MF applied alone or in combination with pterostilbene were applied for 5 consecutive days. Kidney homogenates were analyzed by Fourier transform infrared spectroscopy. I/R injury resulted in an altered protein and lipid structure with the dominance of longer acyl chains; a slight decrease in lipid, protein, unsaturated lipid, and unsaturated/saturated lipid content; and an increase in membrane fluidity and lipid peroxidation in rat kidneys. Although ELF-MF treatment alone was not sufficient to restore all ischemia-induced alterations, the combined treatment strategy of pterostilbene administration in the presence of ELF-MF was successful and warrants further investigation.

## 1. Introduction

Acute renal ischemia-reperfusion (I/R) injury can be described as the damage occurring in ischemic renal cells after the reestablishment of the blood supply (Gao et al-*.*, 2018). Acute renal I/R injury is one of the pathological mechanisms underlying acute renal failure and is likely to occur following renal transplantation and septic shock (Gao et al*.*, 2018). The pathophysiology of acute renal failure has been closely connected to tubular epithelial cell injury. The acute response to renal I/R injury include decrease of both renal blood flow and glomerular filtration rate, as well as reduced tubular function (Allred et al*.*, 2000). I/R injury can be described as the flow of sequential cellular events involving the release of reactive oxygen species, active mediators and formation of free radicals, which result in structural alterations in macromolecules eventually leading to tissue damage, cell apoptosis and necrosis (Fouad et al*.*, 2013; Zou et al*.*, 2013). Nevertheless, renal I/R injury tends to deteriorate over time (Gao et al*.*, 2018)therefore the use of noninvasive treatment strategies will be helpful for the well-being and progression-free survival of the patients.

The use of antioxidants and other agents ha been proposed in the treatment of I/R injuries in the literature (Yamanka et al., 2015; Zhang and McCullough, 2016; Li et al., 2019; Park et al., 2019). Pterostilbene (Pte) is a natural analog of resveratrol ha a higher bioavailability and pharmacokinetic exposure dose in comparison to resveratrol (Kapetanovic et al., 2011). Pte is a well-recognized antioxidant primarily found in blueberries and the heartwood of veving*Pterocarpus marsupium* (Leguminsae). It has traditionally been used for the treatment of diabetes in Indian medicine and has been shown to control blood sugar levels in experimental diabetic animals (McCormack and McFadden, 2013). In addition, Pte has been proposed in recent years to have a promising treatment effect on various disorders such as cancer and nervous system disorders. It was reported to be effective in slowing tumor formation, modulating neurological diseases, decreasing inflammation, reducing symptoms of vascular diseases, increasing cell proliferation in vitro, regulating mitochondrial membrane depolarization, inhibiting caspase activation in vitro or even controlling glucose homeostasis (McCormack and McFadden, 2013). Pte has been reported to enhancea transcription factor activity and mitochondrial function (Pan et al., 2008), attenuate renal injury in septic mice (Xia et al*.*, 2018), reduce oxidative-nitrative stress and inflammatory response uremicseruminduced injury in human umbilical vein endothelial cells (Chen et alon -*.*, 2018). In recent studies the use of Pte was reported to be beneficial in the treatment and prevention of myocardial (Yu et al*.*, 2017), hindlimb (Cheng et al*.*, 2016), cerebral (Zhou et al*.*, 2015) I/R injury. In addition, it was demonstrated to protect acute renal ischemic injury by inhibiting oxidative stress and inflammation (Gao et al*.*, 2018).

Magnetic field is a biophysical agent that has long been proposed as a treatment method for various disorders in the literature, including I/R injury. Some of the reported beneficial effects of extremely low-frequency magnetic field (ELF-MF) include the acceleration of wound healing (Callaghan et al *.*, 2008), enhancement of the rate of bone healing in rats and humans (Grana et al*.*, 2008), modulation of oxygen radicals and changes in signal transduction pathways (Frahm et al*.*, 2000), reduction of cerebral oxidative stress (Rauš Balind et al*.*, 2014). Recently, the use of pulsed magnetic field was suggested to have a role in prevention or treatment of hindlimb (Pan et al*.*, 2013) and myocardial (Ma et al*.*, 2016) I/R injuries. In addition, the application of ELF-MF effectively improved functional and physiological status of ischemic stroke patients (Cichoń et al*.*, 2017). 

However, some disadvantages of the therapeutic application of ELF-MF have also been reported in the literature. Prolonged exposure to ELF-MF was reported to potentially increase plasma levels of proinflammatory cytokines and some hematological parameters (Hosseinabadi et al., 2019), and the application of ELF-MF was demonstrated to induce oxidative stress in rat brain (Manikonda et al., 2014) and to change synaptic plasticity in the hippocampal CA1 regionofuand hindering longterm potentiation in a dose-dependent manner (Zheng et al., 2019). In addition, there have also been some controversial findings in literature related to the effects of ELF-MF on circulation reporting that it may induce (Delle Monache et al*.*, 2008) or inhibit (Delle Monache et al*.*, 2013) cell proliferation and angiogenesis does not affect microvascular responsiveness (McKay et al;*.*, 2010) or inhibit vascular network formation (Costa et al*.*, 2013) exert both positive and negative effects on the development of diabetic nephropathy (Li et al;*.*, 2016). 

Consequently, Pte was previously demonstrated to diminish renal injury in I/R (Gao et al., 2018)however the exact therapeutic potential of ELF-MF application renal I/R injury is still not clear in the literature. In addition, the interaction of ELF-MF with other agents, such as cisplatin, to change its mode of action was previously reported in the literature (Buldak et al., 2012). Accordingly, this study aims to assess the acute therapeutic effects of 50 Hz 1 mT ELF-MF, applied alone or in combination with Pte administration, on macromolecular content, structure and dynamics of kidney macromolecules using Fourier transform infrared (FTIR) spectroscopy in a rat model of renal ischemic damage. FTIR spectroscopy allows the rapid and sensitive characterization of the sample based on macromolecular structure, composition and function by monitoring the alterations in the vibrational modes of functional groups belonging to tissue biomolecules simultaneously (Gasper et al., 2009; Bozkurt et al., 2010, 2012; Baker et al., 2018). Recent studies indicate that FTIR spectroscopy and imaging developing into effective tool in compositional and structural examination of cellular components within intact tissues for diagnostic and treatment monitoring purposes (Gasper et al., 2009; Severcan et al., 2010; Sen et al., 2015; Mordechai et al., 2017; Baker et al., 2018).

## 2. Materials and methods

### 2.1. Reagents

Pte, Silbinol 90% (*Pterocarpus marsupium* extract) standardized for a minimum of 90% terostilbene, was generously provided by Sami Labs Limited (Bangalore, India). Dimetheylsulfoxide (DMSO), sodium dihydrogen phosphate (NaH_2_PO_4_), disodium hydrogen phosphate (Na2HPO4), potassium chloride (KCl), phenyl methyl sulonyl fluoride (PMSF), ethylene diamine tetra acetic acid (EDTA), DL-dithiothreitol (DTT), ethanol w acquired from Sigma (Sigma Chemical Company, St Louis, M, USA) at the highest grade of purity available.

### 2.2. Induction of renal ischemia-reperfusion model and treatment with magnetic field and pterostilbene 

All experimental procedures were conducted according to the approval of the Ethics Committee of Adnan Menderes University with approval number 64583101/2014/068. dult male Wistar rats (300-350 g), obtained from the Center of Experimental Animals of Adnan Menderes University, were fed with a standard diet ad libitum. The animals were divided randomly into 4 experimental groups as control (C) (n = 9), ischemia-reperfusion (I/R) injury (n = 10), ELF-EMF treated I/R injury (MF) (n = 8), combined Pte and ELF-EMF treated I/R injury (MF-P) (n = 7) groups. 

Rats in the control group were not subjected to any operation or treatment. Rats in the I/R, MF, and MF-P groups were anesthetized and subjected to operation for the induction of the I/R injury model as described in the literature (Inal et al., 2002; Nath et al., 2005). Briefly, ischemia was induced by clamping the left renal pedicle for 20 min with a clamping pressure of approximately 10–15 g at a 45° angle using disposable single-use mini bulldog clamps (VASCU-STATT, Scanlan International, USA) under sterile conditions. After induction of ischemia, treatment was started, followed by 24 h of reperfusion.

The rats in MF and MF-P groups were exposed to 50 Hz, 1 mT magnetic field. The parameters of ELF-MF were chosen in accordance with the literature investigating the effect of ELF-MFs on circulation (Dell Monache et al., 2008). The magnetic field exposure was performed by the use of a pair of solenoids placed in a Faraday cage in a room at a controlled temperature (25 ± 2 C). The solenoids were specially to have 1400 turns of insulated 1.4 mm copper wire onto a tubular core cylinder (500 mm in length and 210 mm in diameter). During exposure, an electrical current of 50 Hz, 220 V was passed through the device and the intensity of the magnetic field inside the solenoid was measured with a teslameter (Phywe, Germany), which was observed to be constant. Rats were placed in the middle of solenoids in ventilated Plexiglas cages and exposed to magnetic field daily for an hour (between 9ºdesigned –-12) for 5 consecutive days. The rats in MF-P group also received an intraperitoneal Pte injection at a daily dose of 10 mg/kg, as described previously (Cheng et alAMa.m. in the morning*.*, 2016; Yu et al*.*, 2017), for 5 days. At the end of the fifth day, rats were sacrificed, the kidneys were removed and kept at –80 °C until use. 

### 2.3. Sampling, data collection and analysis for FTIR spectroscopy

After weighing the kidneys, tissues were homogenized (1:4 w/v) in icecold homogenization solution containing 5 mM EDTA, 0.2 mM PMSF, 1.15% (w/v) KCl, 0.2 mM DTT and 0.2 M phosphate buffer (pH 7.4) using a homogenizer (Ultra Turrax-25, IKA, Germany). All steps were performed at 0–4 °C. The homogenates were centrifuged at 4100 rpm for 5 min and the pellets were used for FTIR spectroscopic studies. 

**Figure 1:**
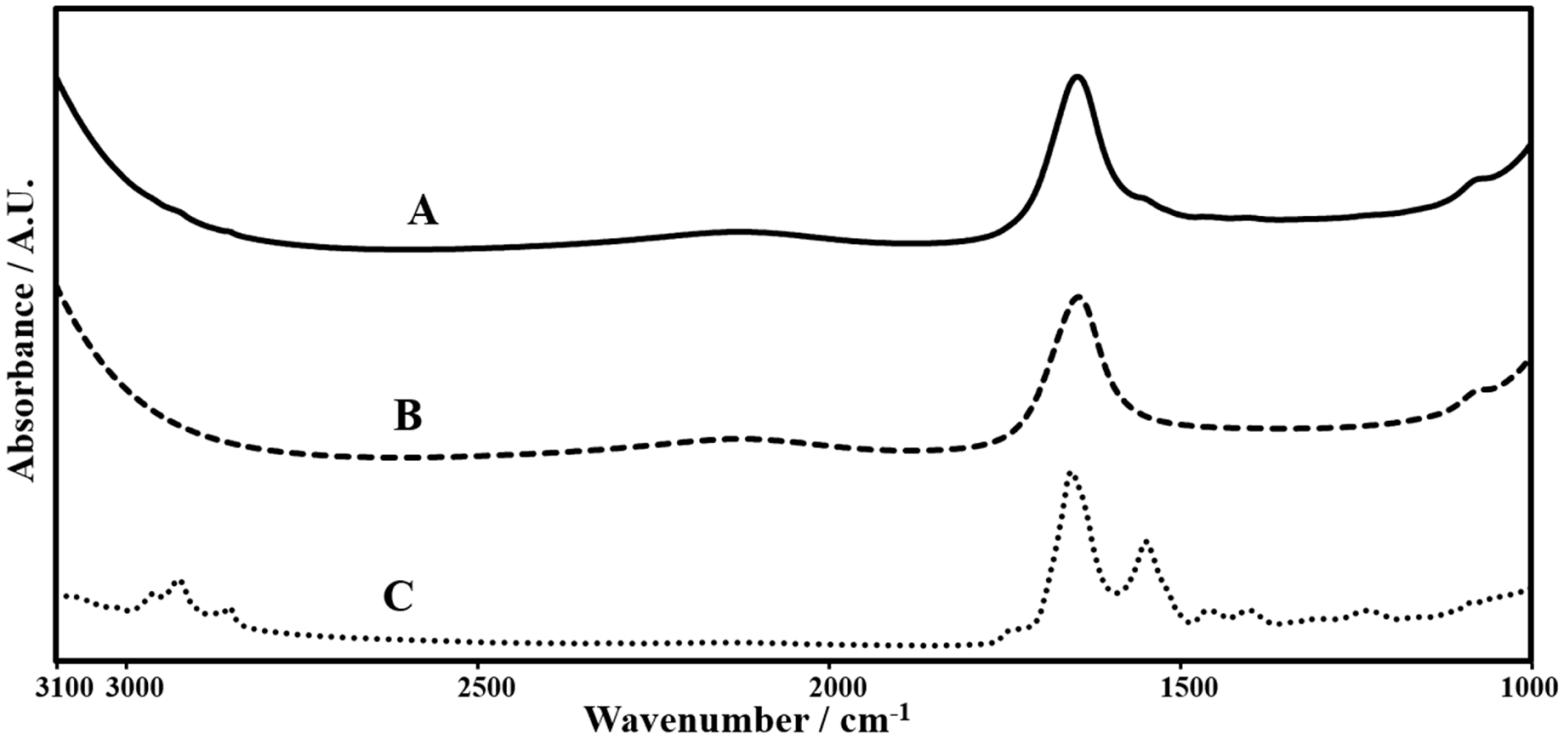
Representative FTIR spectra demonstrating A) control kidney homogenate before subtraction of buffer spectrum, B) buffer spectrum, and C) buffer-subtracted spectrum of control kidney homogenate in the 3100–1000 cm^-1^ frequency range.

Twenty microliters of kidney homogenates was placed between CaFl2 windows with a sample thickness produced by spacers. The spectra of homogenates were recorded in the 4000-650 cm^-1^ region at room temperature by an FTIR spectrometer (PerkinElmer Spectrum 100, Perkin-Elmer Inc., USA) equipped with a MIR TGS detector. Interferograms were acquired the accumulation of 100 scans at 2 cm ^-1^ resolution. The spectra of the samples considered for data analysis were obtained by taking the average of spectra collected from different aliquots of each sample that been scanned under the same conditions. Spectra of buffer solution were also collected using same resolution and scan number. The spectrum of air was collected as background and automatically subtracted from sample and buffer spectra in order to remove the interference of carbon dioxide and water vapor. In detailed data analysis, nonthreehas normalized spectra were analyzed by Spectrum 100 software (PerkinElmer). Firstly, the spectrum of 15 -μ buffer solution, which gave the best result, was subtracted from sample spectra in order to get rid of the interference of water in the sample spectra (Figure 1). The spectra were then smoothed with a 13 point Savitzky-Golay smooth function. The wavenumber corresponding to the center of weight of each band was recorded as the band position, and the width in terms of cm^-1^ at the center of weight of the band was recorded as the bandwidth. Smoothed and baseline-corrected spectra were used to calculate band areas. 

The analysis of the changes in intensities of the subbands of amide I band belonging to protein secondary structures was performed using the OPUS 5.5 data collection software package (Bruker Optics, Germany). For this purpose, second derivative spectra of the 1700-1600 cm^-1^ region were obtained using a 9 smoothing point Savitzky-Golay algorithm and were then vector-normalized in the same range. The peak intensities of the subbands in the 1700-1600 cm^-1^ region were measured from vector-normalized second derivative spectra.

### 2.4. Statistics

The results were stated as mean ± standard error of mean (SE). The Kolmogorov–Smirnov test was used to demonstrate the normal distribution of the data. Statistical analysis of differences in variance was then performed by one-way analysis of variance (ANOVA) followed by Tukey’s test as a post hoc test. P ≤ 0.05 was accepted as statistically significant (*P < 0.05, **P < 0.01, ***P < 0.001). The degree of significance was symbolized with asterisks (*), double daggers (‡), and daggers (†) for the comparisons of control, I/R, and MF groups, respectively, with other groups.

## 3. Results 

**Figure 2:**
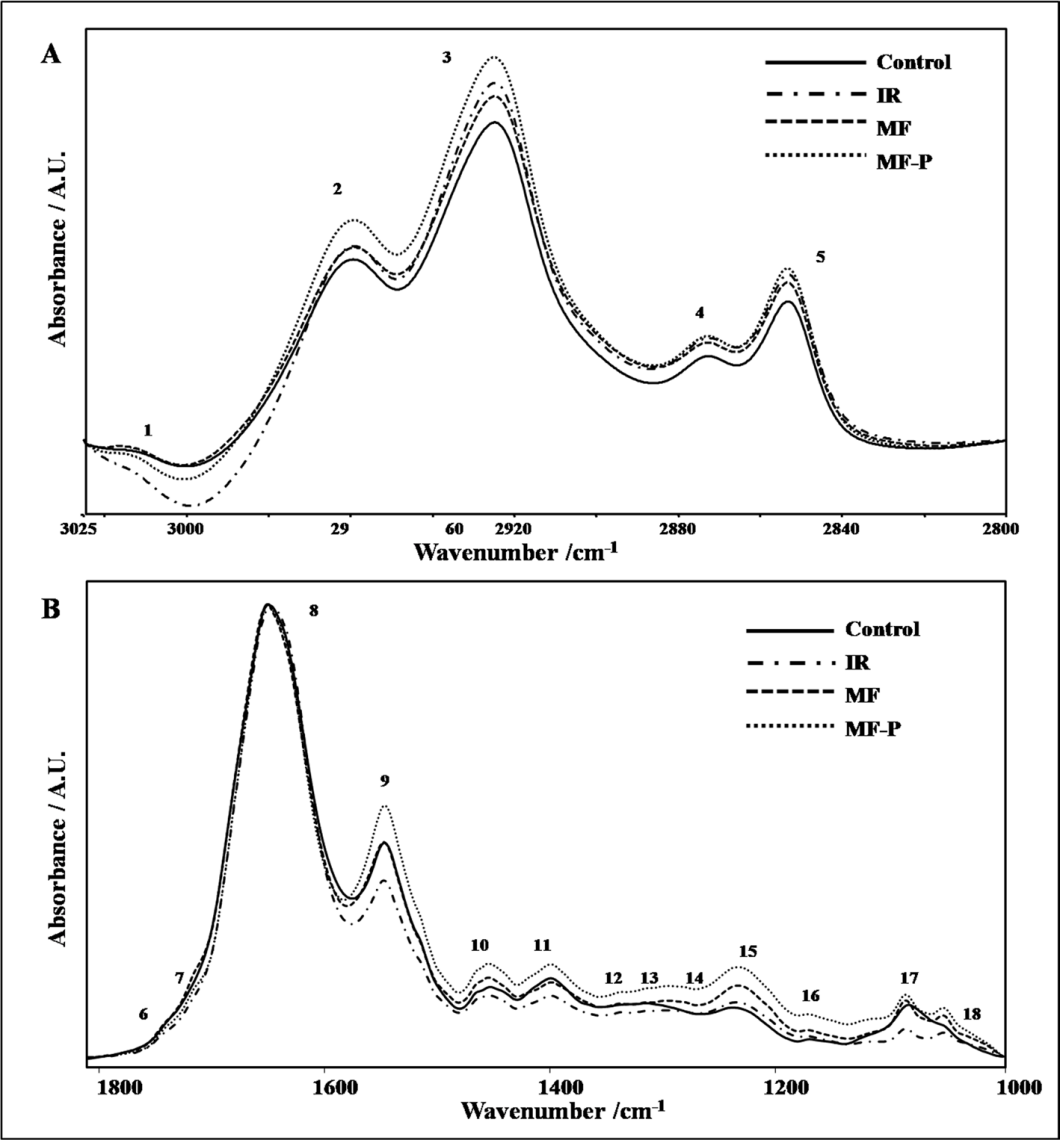
Representative FTIR spectra of kidney homogenates of control, ischemia-reperfusion (I/R), I/R treated with magnetic field (MF), and I/R received combined treatment of magnetic field and 10 mg/kg Pte (MF-P) groups in the A) 3025–2800 cm^-1^ and B)1800–1000 cm^-1^ frequency range. Spectra were normalized with respect to the amide I band.

Vibrations of different functional groups in the structure of molecules give rise to characteristic absorption bands at a particular frequency in the FTIR spectrum (Umemura et al*.*, 1980; Bozkurt et al*.*, 2010; Ozek et al*.*, 2010; Cakmak et al*.*, 2011). Figures 2A and 2B show the representative FTIR spectra of kidney homogenates of the experimental groups in the frequency ranges of 3025-2800 cm^-1^ and 1800-1000 cm^-1^ , respectively. The main absorption bands are labeled in the figures and the definitions of the bands re given in Table 1. Table 2 shows the changes in frequency, band area and bandwidth values of some of the absorption bands in the spectra of the experimental groups. 

**Table 1:** The assignments of the major bands in the infrared spectra of kidney homogenates in the 3100–1000 cm^-1^ region.

Bandno.	Wavenumber(cm^-1^ )	Definition
1	3014	Olefinic = CH: Unsaturated lipids (Sen et al., 2015; Cakmak et al., 2011; Movasaghi et al., 2008)
2	2962	CH_3_ antisymmetric stretching: Lipids and protein side chains (Szalontai, 2009; Severcan et al., 2010; Sen et al., 2015)
3	2925	CH_2_ antisymmetric stretching: Mostly lipids (Cakmak et al., 2011; Severcan et al., 2010; Sen et al., 2015)
4	2873	CH_3_ symmetric stretching: Mostly proteins (Cakmak et al., 2011; Severcan et al., 2010; Sen et al., 2015)
5	2854	CH_2_ symmetric stretching: Mostly lipids (Cakmak et al., 2011; Sen et al., 2015)
6	1738	Ester C=O stretching: Lipid, cholesterol ester groups (Bozkurt et al., 2012; Nara et al., 2002)
7	1713	C=O stretching: Free fatty acids (Nara et al., 2002)
8	1650	Amide I: Protein, 80%, C=O stretching, 10%, N-H bending, 10%, C-N stretching (Haris and Severcan, 1999)
9	1545	Amide II: Protein, 60%, N-H bending, 40%, C-N stretching (Haris and Severcan, 1999)
10	1455	CH_2_ bending: Mostly lipids (Cakmak et al., 2011; Ozek et al., 2010; Sen et al., 2015)
11	1401	COO- symmetric stretching: Fatty acids and protein side chains (Bozkurt et al., 2012)
12	1340	CH_2_ side chain vibrations of collagen (Movasaghi et al., 2008; Bozkurt et al., 2010)
13	1317	Amide III: Protein side components, collagen (Movasaghi et al., 2008)
14	1283	Collagen (Movasaghi et al., 2008)
15	1234	PO2- antisymmetric stretching: Mostly nucleic acids, small amounts of phospholipids (Ozek et al., 2010)
16	1170	CO-O-C antisymmetric stretching: Ester bonds of cholesterol esters (Ozek et al., 2010; Sen et al., 2015)
17	1084	PO2- symmetric stretching: Nucleic acids and phospholipids (Bozkurt et al., 2010; Movasaghi et al., 2008)
18	1049	νC–O + δC–O stretching: Glycogen (Bozkurt et al., 2012; Sen et al., 2015)

**Table 2:** Changes in the frequency, band area, and bandwidth values of some of the absorption bands in the kidney FTIR spectra of control, ischemia-reperfusion (I/R), I/R treated with magnetic field (MF), and I/R treated with combined magnetic field and 10 mg/kg Pte (MF-P) groups.

Band no.	Wavenumber(cm^-1^ )	Control (n = 9)	I/R (n = 10)	MF (n = 7)	MF-P (n = 8)
Band frequency					
2	2962	2960.894 ± 0.29	2961.137 ± 0.40	2963.666 ± 0.88**,††	2960.836 ± 0.06‡‡
3	2925	2922.579 ± 0.06	2922.7 ± 0.05	2922.774 ± 0.08	2922.689 ± 0.05
5	2854	2852.20 ± 0.08	2852.44 ± 0.06	2852.46 ± 0.06	2852.54 ± 0.06*
Band area					
1	3014	3.94 ± 0.74	2.52 ± 0.33	4.00 ± 0.55	4.23 ± 0.56
2	2962	3.98 ± 0.55	2.85 ± 0.31	4.06 ± 0.48	4.27 ± 0.46
3	2925	3.99 ± 0.39	3.20 ± 0.28	5.05 ± 0.52†	4.02 ± 0.36
4	2873	0.75 ± 0.07	0.82 ± 0.08	0.85 ± 0.08	0.78 ± 0.06
5	2854	1.02 ± 0.09	0.83 ± 0.07	1.17 ± 0.12	1.19 ± 0.10
8	1650	31.69 ± 5.76	28.22 ± 2.67	37.31 ± 4.65	33.89 ± 3.32
9	1545	8.95 ± 0.93	8.85 ± 0.80	12.71 ± 1.48	9.20 ± 0.59
15	1234	1.67 ± 0.23	1.87 ± 0.23	2.97 ± 0.44*	1.92 ± 0.19
18	1049	0.23 ± 0.04	0.34 ± 0.06	0.63 ± 0.11**,†	0.70 ± 0.07***,††
Bandwidth					
2	2962	10.95 ± 1.44	11.63 ± 0.66	9.69 ± 0.18	9.22 ± 0.83
3	2925	9.88 ± 0.35	11.63 ± 0.12***	9.42 ± 0.19†††	9.64 ± 0.14†††
5	2854	9.84 ± 0.37	10.79 ± 0.53	9.62 ± 0.25	10.20 ± 0.27

The data were represented as mean ± standard error of mean. Comparison was performed by the one-way ANOVA test with Tukey’s test as a post hoc test. The degree of significance was denoted as *P < 0.05, **P < 0.01, and ***P < 0.001. The degree of significance was denoted by * for the comparison of the control with the other groups, by ‡ for the comparison of the I/R group with the other groups, and by † for the comparison of the MF group with the other groups.

In addition, band area ratios were used to monitor the alterations in hydrocarbon chain lengths of lipidsthe amount of proteins and lipids in tissues’,the amount of carbonyl, ethyl and methyl groups in lipidsthe amount of unsaturation in lipidsthe structural changes in the tissue proteins and metabolic activity (Bozkurt et al., 2010; Cakmak et al., 2012; Sen et al*.*, 2015) in kidney tissues (Table 3). The total lipid amount was calculated by taking the sum of the band areas of the olefinic (HC = CH), the CH_2_ antisymmetric stretching, the CH_2_ symmetric stretching, the CH_3_ antisymmetric stretching, the COO-symmetric stretching, the CO-O-C antisymmetric stretching and the CH_2_ bending vibrations, all of which originat from lipids in the system.

**Table 3:** Changes in some band area ratio values in the kidney FTIR spectra of control, ischemia-reperfusion (I/R), I/R treated with magnetic field (MF), and I/R treated with combined magnetic field and 10 mg / kg Pte (MF-P) groups.

Calculated ratio	Control(n = 9)	I/R(n = 10)	MF(n = 7)	MF-P(n = 8)
Total lipid/amide II (total lipid/protein)	1.75 ± 0.13	1.34 ± 0.05	1.42 ± 0.06	1.83 ± 0.18†
CH_2_ sym. str./CH_3_ sym. str. (lipid/protein)	1.37 ± 0.03	1.01 ± 0.01***	1.38 ± 0.01†††	1.53 ± 0.02***,†††,‡‡
CH2 antisym. str./CH3 antisym. str. (hydrocarbon chain length)	1.08 ± 0.08	1.15 ± 0.04	1.26 ± 0.03	0.96 ± 0.03‡‡
CH_3_ antisym. str./total lipid (amount of methyl groups in the lipids)	0.25 ± 0.006	0.24 ± 0.003	0.23 ± 0.003**	0.25 ± 0.004†, ‡‡‡
CH2 antisym. str./total lipid (amount of ethyl groups in the lipids)	0.26 ± 0.013	0.27 ± 0.007	0.28 ± 0.004	0.25 ± 0.004
C=O ester str./total lipid (amount of carbonyl group in lipids)	0.04 ± 0.002	0.04 ± 0.002	0.04 ± 0.003	0.04 ± 0.03
Olefinic=CH/CH_2_ antisym. str. + CH_2_ sym. str.(unsaturated/saturated lipid ratio)	0.74 ± 0.10	0.62 ± 0.05	0.63 ± 0.03	0.80 ± 0.07
Olefinic=CH/total lipid (unsaturated lipid content)	0.23 ± 0.021	0.21 ± 0.011	0.22 ± 0.007	0.25 ± 0.012
Amide I/amide II (structural change in proteins)	3.37 ± 0.38	3.21 ± 0.18	2.92 ± 0.11	3.66 ± 0.21
νC–O + δC–O stretching/PO2- sym. str. (metabolic activity)	0.64 ± 0.30	3.40 ± 0.83	2.62 ± 0.43	1.49 ± 2.28

The data were represented as mean ± standard error of mean. Comparison was performed by the one-way ANOVA test with Tukey’s test as a post hoc test. The degree of significance was denoted as *P < 0.05, **P < 0.01, and ***P < 0.001. The degree of significance was denoted by * for the comparison of the control with the other groups, by ‡ for the comparison of the I/R group with the other groups, and by † for the comparison of the MF group with the other groups.

### 3.1. Proteins

There was a slight decrease in the area of amide I and II bands, which originate from proteins in the system, in I/R injury group and the protein content was found to be slightly increased in treatment groups (Table 2). mide I/amide II ratio, obtained by the taking the ratio of amide I band area divided by amide II band area, can be used to determine changes in protein structure (Bozkurt et alA*.*, 2012). According to the results obtained, amide I/amide II ratio decreased slightly in I/R injury and magnetic field treatment groups and increased in combined Pte and magnetic field treatment group (Table 3). 

**Figure 3:**
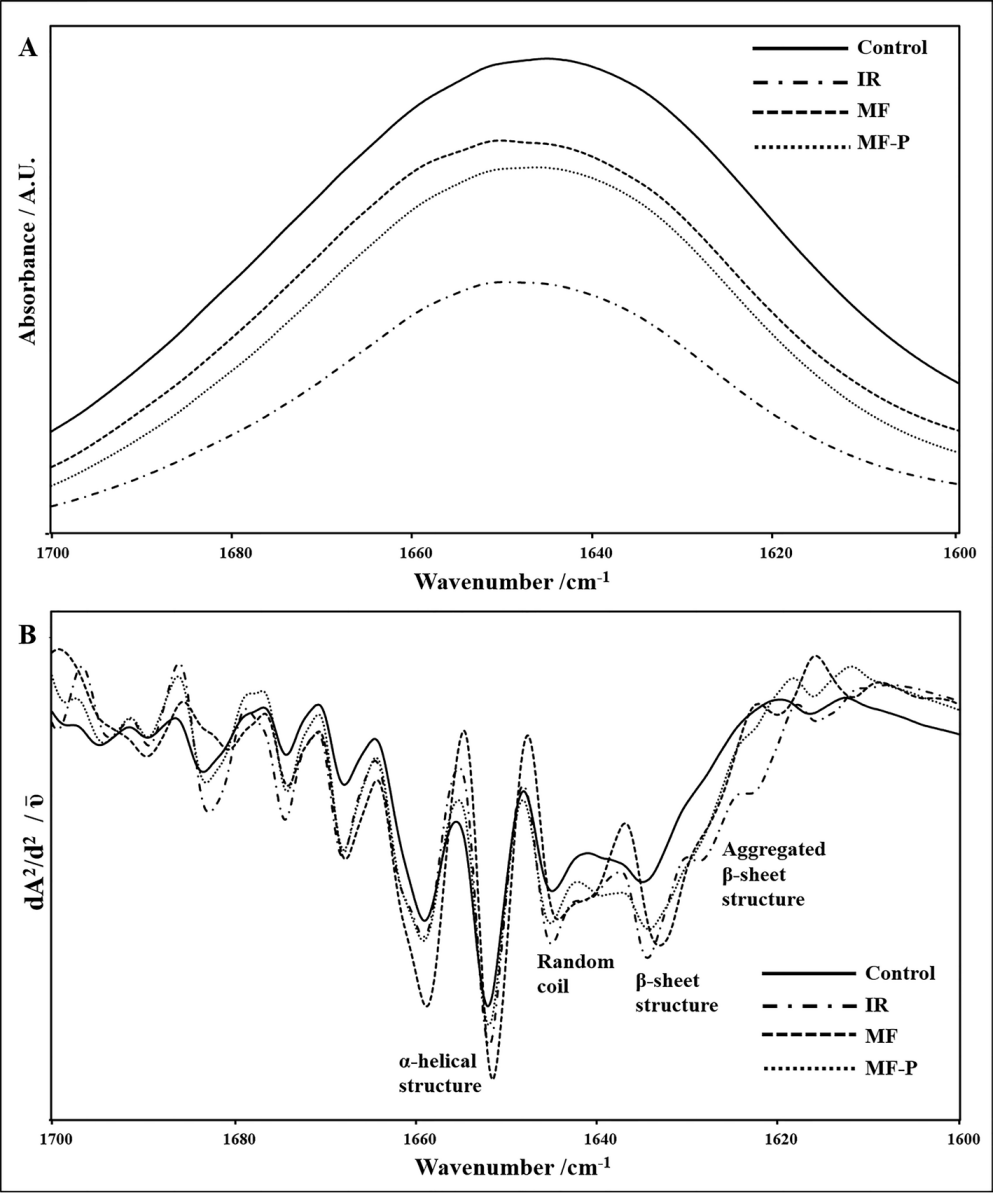
Representative (A) absorbance FTIR spectra and (B) second derivative spectra of the amide I band for control, ischemiareperfusion (I/R), I/R treated with magnetic field (MF), and I/R received combined treatment of magnetic field and 10 mg/kg Pte (MFP) kidney homogenates in the 1700–1600 cm^-1^ region. Vector normalization was performed in the 1700–1600 cm^-1^ region. Absorption maxima appear as minima in the second derivatives.

These results suggested a slight change in protein secondary structure in kidney homogenates, and the analysis of the intensities of the subbands in the second derivative FTIR spectra (Figure 3; Table 4) also revealed a slight change in protein secondary structures. The intensity of the α-helix subband located at 1658 cm^-1^ decreased slightly in the kidneys of the I/R injury group, together with a slight increase in the intensity of the β-sheet subband located at 1637 cm^-1^. Both treatments led to an increase in the intensity of the α-helix subband and a decrease in the intensity of the β-sheet subband, although the change was not statistically significant. The intensities of the subbands of the aggregated β-sheet and random coil are indicative of protein denaturation (Bozkurt et al., 2010). The intensities of the subbands of the aggregated β-sheet located at 1628 cm^-1^ and the random coil located at 1645 cm^-1^ (Bozkurt et al., 2010) increased slightly in the kidneys of the I/R injury group, and their intensities were decreased upon both treatments. 

**Table 4:** The results of the changes in the intensities of main functional groups of the second derivative FTIR data in 1600–1700 cm^-1^ spectral region (amide I band) representing the alterations in protein secondary structure for kidneys of control, ischemia-reperfusion (I/R), I/R treated with magnetic field (MF), and I/R treated with combined magnetic field and 10 mg/kg Pte (MF-P) groups. The data were represented as mean ± standard error of mean.

Functional groups	Control (n = 9)	I/R (n = 10)	MF (n = 7)	MF-P (n = 8)
α-Helical structure (at 1658 cm^-1^ )	–0.162 ± 0.020	–0.134 ± 0.017	–0.156 ± 0.005	–0.141 ± 0.014
β-Sheet structure (at 1637 cm^-1^)	–0.115 ± 0.006	–0.125 ± 0.009	–0.108 ± 0.003	–0.119 ± 0.003
Aggregated β-sheet structure (at 1628 cm^-1^)	–0.066 ± 0.005	–0.078 ± 0.009	–0.056 ± 0.012	–0.065 ± 0.010
Random coil (at 1645 cm^-1^)	–0.093 ± 0.009	–0.105 ± 0.012	–0.079 ± 0.005	–0.100 ± 0.006

### 3.2. Lipid to protein ratio

The lipid to protein ratio in an FTIR spectrum can be used to visualize the relative amount of proteins and lipids in the system, which is one of the most important influences on membrane structure and dynamics (Szalontai et al**:**

*.*, 2000; Bozkurt et al*.*, 2010). ipid to protein ratio was calculated by taking the ratio of total lipid band areas divided by the area of amide II band and by taking the ratio of the area of the CHL;2 symmetrical stretching band (lipids) to the area of the CH3 symmetrical stretching band (proteins) (Table 3). The lipid to protein ratio significantly decreased in the I/R injury group in comparison to the control, while both treatments led to a significant increase in the lipid to protein ratio in kidney homogenates in comparison to I/R group, most prominently in the MF-P group.

### 3.3. Lipids

In addition to protein content, the areas of the lipid bands slightly decreased in the I/R injury group revealing a decreased lipid content, whereas the treatments resulted in a slight increase in the kidney lipid content (Table 2). 

The olefinic band (HC=CH) located at approximately 3014 cm^-1^ in an FTIR spectrum originates from unsaturated HC=CH vibrations in the lipids of the sample and can be used to monitor lipid peroxidation (Bozkurt et al*.*, 2010; Ozek et al*.*, 2010; Sen et al*.*, 2015). Besides, the amount of unsaturated/saturated lipids (unsaturation index) can be monitored in FTIR spectrum by using the ratio of the olefinic (HC=CH) band area (unsaturated lipids) to the CH_2_ antisymmetric and symmetric stretching band areas (saturated lipids) and the rate of unsaturation can be determined by the olefinic (HC=CH)/total lipid band area ratio (Gasper et al., 2009; Cakmak et al., 2011, 2012; Sen et al., 2015). As s by the results obtained in this study, the area of the olefinic band decreased in the I/R injury group and both treatments led to an enhancement in the olefinic band area (Table 2). Moreover, the unsaturated/saturated lipid ratio and the unsaturated lipid content of lipids (Table 3) also decreased in h Cakmak et al*.*tatedwereI/R injury group and increased upon treatment, especially the combined Pte and magnetic field treatment.

The band positions of the CHwereby3 antisymmetric, the CH2 antisymmetric and symmetric stretching bands can be used to assess the degree of the elasticity of the lipid acyl chains and the conformational disorder of the lipidshence they can be used as a marker to examine the average trans/gauche isomerization in the system (Bozkurt et al., 2010, ). Shift to higher values seen in the frequencies of these bands show the increase in gauche conformers and the disorder of acyl chains in the system (Severcan et al., 2005; Bozkurt et al., 2010; Cakmak et al., 2011).; Severcan et al*.*, 2005ingsOzek et al., 2010; Moreover, the bandwidth of these bands gives information about fluidity and dynamics of the membrane (Severcan et al., 2005; Bozkurt et al., 2010). An increase in bandwidth values implies increase in membrane fluidity (Severcan et al., 2005). In addition, the change in the CHthe3 antisymmetric stretching band frequency values give information about the order/disorder state of the inner membrane area, as CH3 moieties reside in the deep interior of the membrane (Umemura et al., 1980). There was a shift to higher values in the frequency of the CH3 antisymmetric stretching band in I/R injury and magnetic field treatment groups (Table 2), revealing the increase in disorder of inner membrane areas. , the combined treatment of Pte and magnetic field restored the alteration in membrane disorder and resulted in the shift of the frequency of this band to lower values as the control group. No significant changes were observed in the frequencies of the CHYetthat of2 antisymmetric stretching vibrations (Table 2). However, bandwidth values of the CH_2_ antisymmetric stretching vibration were increased in the I/R injury group, which shows an increase in lipid fluidity and this increase was restored to that of control values in the treatment groups.

In order to evaluate the alterations in lipid structure, parameters such as the hydrocarbon chain length and the amount of carbonyl, ethyl and methyl groups in lipids can be used in an FTIR spectrum (de Zwart et al., 1999; Cakmak et al., 2012; Sen et al., 2015). The CH_3_ antisymmetric stretching/total lipid and the CH_2_ antisymmetric stretching/total lipid band area ratios can be used to observe the alterations in the amount of methyl and ethyl groups in the lipids, respectively (Cakmak et al., 2011, 2012). It is known that the hydrocarbon chains of the lipids contain CH_2_ groups at the trunk portion and CH_3_ molecules at the tip portion. The CH_2_ antisymmetric stretching/CH_3_ antisymmetric stretching band area ratio in the FTIR spectrum gives information about the lipid hydrocarbon chain length (Gasper et al., 2009). This ratio was observed to increasebe slightly in the I/R injury group and the magnetic field treatment group, revealing that the length of hydrocarbon chains in lipids tend to be longer in these groups. The combined Pte and magnetic field treatment led to a significant decrease in this ratio, suggesting that shorter lipid acyl chains were observed in the combined treatment group. These results were also supported by the increase in ethyl and the decrease in methyl groups in lipid structures of I/R injury and magnetic field treatment groups (Table 3). The combined Pte and magnetic field treatment restored these alterations in ethyl and methyl group content in lipid structures of kidneys. No significant changes were observed in the carbonyl content of lipids between the groups, when the C=O ester stretching/total lipid ratio, indicative of the amount of carbonyl groups in lipid structures in the membranes (Cakmak et al., 2011, 2012), was examined (Table 3). 

### 3.4. Carbohydrates

The area of the νC–O + δC–O stretching band (1049 cm^-1^), which originates from glycogen and carbohydrate molecules in the system (Sen et al., 2015), was observed to be significantly increased in treatment groups (Table 2). In order to better comment on this result, glycogen/phosphate ratio, can be used to monitor metabolic activitythat (Ozek et al., 2010; Sen et al., 2015), was calculated by taking the ratio of the area of the νC-O + δC-O stretching band divided by the area of the PO2- symmetrical stretching band, which originates mainly from nucleic acids with a contribution from the head groups of phospholipids in membranes. The glycogen/phosphate ratio was found to be slightly increased in I/R injury group and slightly decreased in treatment groups (Table 3).

## 4. Discussion

Although the effective role of Pte administration in preventing I/R injury was previously reported (Gao et al., 2018), the potential efficacy of ELF-MF in renal ischemic injury is still not clear and there have been controversial results reported in the literature regarding the efficiency of ELF-MF. Accordingly, this study was conducted to assess the therapeutic potential of continuous 50 Hz, 1 mT ELF-MF, applied alone or in combination with Pte, in a rat model of renal I/R injury. FTIR spectroscopy was used as a reliable and efficient method for the study of structural, compositional and functional characterization of the biomolecules in kidney homogenates. In an FTIR spectrum, information on the concentration or content of a particular functional group can be acquired from band areas and/or signal intensities information about structural alterations in macromolecules can be obtained by monitoring the frequency shifts of the bands and bandwidth values can be used to monitor dynamic properties of various molecules in the system (Umemura et al., 1980; Bozkurt et al., 2010; Cakmak et al., 2011, 2012). 

Lipids are the major constituents of the cell membrane, and alterations in lipid structure and content may lead to membrane dysfunction. Lipid peroxidation is a cellular damage mechanism triggered by free radicals, which is indicative of elevated oxidative stress in the system. The attack of free radicals on unsaturated bonds of lipids results in a reduced number of unsaturated bonds in the lipid content (de Zwart et al., 1999). It has been documented that free oxygen species are elevated in ischemia resulting in damage in lipids (Zou et al., 2013). In accordance with the literature, the results of the current study revealed that I/R injury led to a decrease in the amount of unsaturated lipids demonstrating an increase in lipid peroxidation in kidney tissues, and the treatments, more prominently the combined treatment were effective in the reduction of the lipid peroxidation. In addition, I/R led to a slight decrease in protein content and a slight decrease in lipid content together with a significant decrease in the lipid to protein ratio, that the decrease in lipid content was much more prominent in comparison to the decrease in protein content in I/R injury. I/R injury also led to changes in lipid structure and chain length, where the dominanc of lipids with longer acyl chains w observed in I/Rinjured kidney homogenates. Both treatments, especially the combined Pte and magnetic field treatment, successfully restored the I/Rinduced alterations in lipid and protein content and restored the alterations in lipid structure and chain length. The reason for these alterations in lipid structure may be lipid peroxidation, since the attack of free radicals may lead to changes in chemical bonds of lipids and lipid structure (de Zwart et al.,1999). In addition, renal I/R injury and especially ELF-MF applicationimplicating yere, resulted in the increase in disorder of inner membrane areas revealed by the shift of the frequency values of the CH_3_ antisymmetric stretching vibration to higher values. However, no alteration was observed in the frequency of CH_2_ antisymmetric stretching bands between the groups. The membrane fluidity was observed to be increased in renal I/R group, which was restored to that of control upon treatments.

The reported results in the literature demonstrate that the frequency and the flux density of the applied magnetic field or the mode of application influenc the effects of magnetic field on lipid peroxidation. It was reported that 60 Hz frequency electromagnetic field induced no changes in the concentration of thiobarbituric acid reactive productssare ing which is a lipid peroxidation indicator, in liver, heart, kidney and plasma of Wistar rats (Martínez-Sámano et al., 2010). However, 40 Hz low-frequency 10 mT electromagnetic field stimulated glutathione peroxidase activity, inhibited phospholipid peroxidation and as a result stabilized cellular membranes (Glinka et al., 2013). Lipid peroxidation was significantly increased in rat livers exposed to 4.7 T static magnetic field for 3, 6, 24, and 48 h, but no changes wours observed in kidney, heart, lung, and brain tissues (Watanabe et al., 1997). However, pulsed magnetic field of 1.5 mT was observed to be protective against lipid peroxidation in the liver (Emre et al., 2011).as In the present study, the continuous magnetic field of 50 Hz 1 mT applied 1 h per day for 5 consecutive days reduce the lipid peroxidation in I/Rinjured kidneys. However, magnetic field application alone was not as effective as the combined 10 mg/kg Pte and magnetic field therapy in preventing ischemiainduced alterations in lipid structure.

The increase in reactive oxygen species in a system not only results in lipid peroxidation,our,s but also leads to changes in protein structure such as tertiary structure changes, reduction, degradation and peroxidation, resulting in indirect or direct damage proteins (Kohen and Nyska, 2002; Khan et al., 2015). The frequency of the amide I vibration in an FTIR spectrum is very sensitive to conformational alterations in proteins; therefore, amide I band can be used to determine changes in protein secondary structure by the use of second derivative spectra (Haris and Severcan, 1999; Bozkurt et al., 2010; Rieppo et al., 2012; Talari et al., 2017). The results implicated a slight alteration in protein secondary structure by a slight decrease in the intensity of the α-helix subband together with a slight increase in the intensities of the β-sheet, aggregated β-sheet and random coil subbands in kidney homogenates of I/R injury group (Table 4). Both treatments led to an increase in the intensity of ,in--α-helix subband and a decrease in the intensities of β-sheet, aggregated β-sheet and random coil subbands, although the change was not statistically significant. Therefore, I/R injury led to slight changes in protein secondary structure, which was restored upon treatment. 

The glycogen/phosphate ratio, which the relative changes in glycogen and phosphate groups, can be used to monitor the alterations the usage of carbohydrate sources, the rate of glycogen and/or carbohydrate conversion and the amount of metabolic turnover of the carbohydrate sources for energy production (Ozek et al., 2010; Sen et al., 2015). A decrease in this ratio suggests an increase in metabolic turnover of carbohydrates or a higher metabolic activity (Ozek et al., 2010; Sen et al., 2015). The increase in the glycogen/phosphate ratio in I/R injury group and the decrease in treatment groups revealed that the metabolic activity and turnover of the carbohydrate sources decrease with ischemic injury--sdisplays on s and can be increased especially by the combined Pte and magnetic field treatment. Renal cell turnover is reported to change in different pathological conditions, but generally proliferation of proximal tubule cells was stated to be nearly completed 7 days an induced renal injury (Fujigaki, 2012). In addition, Duffield indicated that necrotic intratubular debris was widespread 2 days after renal ischemic injury, but tubular architecture ,afterofand coworkersthe was significantly restored and the plasma creatinine levels were completely recovered 7 days renal ischemic injury (Duffield et al., 2005). Our results of decreased metabolic turnover at the 5th day ischemic injury could haveafterofpost- resulted from the necrosis occurred during the I/R procedure however, the treatments, especially the combined treatment, w able to increase the metabolic turnover in kidneys.

Previous studies have reported detrimental, beneficial or ineffective actions of electromagnetic field on biological systems, mainly depending on the frequency and the flux density of the magnetic field together with the mode and duration of administration. In the literature, 50 Hz, 2 mT sinusoidal magnetic field was reported to inhibit the formation of new blood vessels by exerting antiangiogenic effects on the vascular endothelial growth factor signaling pathwaybeen,as- and was suggested as a therapeutic treatment for angiogenesis-related diseases such as cancer, ischemic diseases and chronic inflammation (Delle Monache et al., 2013). However, in another study of the same group, 50 Hz, 1 mT sinusoidal magnetic field application was observed to enhance angiogenesis (Delle Monache et al., 2008). ulsed magnetic field was observed to have a role in wound healing by increasing perfusion and vascularization (Pan et al., 2013) and by increasing endogenous fibroblast growth factor release (Callaghan et al., 2008). For the treatment of ischemic injury, the application of pulsed and static magnetic field was documented to suppress the oxidative stress and thereby be a possible candidate for the treatment of myocardial (Ma et al., 2016), hindlimb (Pan et al., 2013) ischemic injuries and the rehabilitation of stroke patients (Cichoń et al., 2017). 

In this current study, the combined treatment strategy of Pte administration in presence of ,P,thepost-50 Hz, 1 mT ELF-MF was observed to be more efficient in ischemia-induced structural and functional alterations in kidneys in comparison to treatment with ELF-MF alone. In the literature, Pte has been shown to possess antioxidant and antiinflammatory actions and was found to be beneficial in treatment of ischemic damage in various tissues (Zhou et al., 2015; Cheng et al., 2016; Yu et al., 2017; Gao et al., 2018). Considering that the application of magnetic field increases vascular permeability (Callaghan et al., 2008; Delle Monache et al., 2013; Pan et al., 2013) and antioxidant enzyme activities (et al., 2010; Glinka et al., 2013), the applied magnetic field in the combined treatment group may have contributed to the observed beneficial effects by increasing the efficacy of endogenous antioxidants and by facilitating the delivery of Pte to damaged cells in the affected kidneys. In conclusion, the combined strategy of Pte treatment in the presence of magnetic field was observed to be a promising method and warrants further investigation.

## Acknowledgments

This work was supported by Adnan Menderes University Research Fund under grant number TPF-15031. Silbinol 90% (the *Pterocarpus marsupium* extract) standardized for a minimum of 90% pterostilbene was generously provided by Sami Labs Limited, Bangalore, India. The authors hereby report that there are no conflicts of interest to declare.
